# Learning from patients' written feedback: medical students' experiences

**DOI:** 10.5116/ijme.61d5.8706

**Published:** 2022-01-31

**Authors:** Karin Björklund, Terese Stenfors, Gunnar Nilsson, Charlotte Leanderson

**Affiliations:** 1 Department of Neurobiology, Care Sciences and Society, Karolinska Institutet, Stockholm, Sweden; 2 Department of Learning, Informatics, Management and Ethics (LIME), Division for learning, Karolinska Institutet, 171 77, Stockholm, Sweden

**Keywords:** Medical students, patient-centred communication, patients-feedback, questionnaire

## Abstract

**Objectives:**

This study explores students' experiences of learning based on patients' written feedback, obtained through the Patient Feedback in Clinical Practice (PFCP) questionnaire.

**Methods:**

Fifty-nine medical students evaluated their learning experience of receiving patients' written feedback obtained from the PFCP questionnaire. Students (N = 57) evaluated their experiences by applying a nine-question evaluation survey (Likert scale N = 3 and free-text questions N = 6) and/or participated in a semi-structured interview (N = 6 students). Data were analyzed using descriptive statistics and qualitative content analysis.

**Results:**

The analysis of data from the students' evaluation survey was performed using 4-point Likert scale questions presented in mean, SD and range; ability to apply patient-centred communication (3.3, 0.74, 2–4), guidance for further clinical training of clinical skills (3.2, 1.31, 1–4) and visualization of the pedagogical assignment during an encounter (3.0, 1.68, 1–4). A content analysis of the free-text questions from the students' evaluation surveys and interviews resulted in three themes: (1) confidence in clinical practice, (2) application of patient-centred communication and (3) identification of learning needs.

**Conclusions:**

The results indicate that patients' feedback facilitated a reflective self-directed learning process with the identification of learning needs and increased awareness of the patient as a collaborative partner during the encounter. Patients' written feedback adjacent to a patient encounter is identified as a valuable additional learning tool in medical students' workplace learning. Further studies are required to explore how patients' written feedback can be operationalized in different clinical contexts, for example, in in-patient care.

## Introduction

For medical students, the ability to communicate and apply patient-centered working methods are core competencies to train and progressively develop.^1^^-^^4^ Feedback is an important tool and a strong motivator that can be used to provide information about how to identify and narrow the gap between the current and desired level of performance during students' workplace learning.^5^^-^^10^ The necessity for students to continuously receive feedback during clinical education is described by previous authors as crucial for students' workplace learning.^11^ Various factors can act as barriers for the provision of feedback, such as time,^11^^-^^14^ and the ability to provide feedback within an adequate time frame for the student to have the ability to act upon.^10^ Other factors related to adequate and successfully provided feedback are, e.g., target specificity, interpretability and contextualization which has been addressed in previous research.^9^^,^^15^

Research shows that patients can provide valuable information and perspectives during students' workplace learning.^16^ Relating the patient's feedback to the student's pre-understanding and the student's own experience from a specific encounter can provide additional pedagogical value for students' workplace learning.^17^ In addition to feedback from clinical supervisors, who traditionally have assessed the students level and competence of applied patient-centredness, patients' feedback can be a valuable contribution.^5^^,^^18^ However, patients are seldom directly involved in students' learning as providers of written feedback.^5^^,^^6^ Previous research indicate that medical students often experience patient feedback as encouraging, moderate and positive^6^^,^^18^^,^^19^ which can be gratifying to receive but may hence not be recognized as an actionable learning tool.^15^

One way for patients to provide feedback as a source of information in students workplace learning is by the use of a feedback questionnaire. In a previous study, we developed a questionnaire for patient's feedback to medical students, the patients' feedback in Clinical Practice (PFCP) questionnaire, for provision of feedback adjacent to a patient encounter.^20^ In the scientific literature, medical students' experiences with direct feedback from patients have received little attention, despite evidence of the importance of including patients as learning partners in students' education.^21^

Therefore, the current study aims to explore medical students' learning experiences from patients' written feedback obtained through the PFCP questionnaire.

## Methods

### Study design and participants

This study was conducted at the undergraduate medical programme at Karolinska Institutet (KI) in Stockholm, Sweden. Data was collected during the students' workplace learning at eight primary health care (PHC) centres, including areas with different socioeconomic status in Region Stockholm. The study explores students' experiences of learning from patients' written feedback, obtained from the PFCP questionnaire,^20^ by use of an evaluation survey and semi-structured interviews. A descriptive statistical analysis of the students' evaluation survey (4-point Likert scale) was performed. Content analysis of the students' evaluation surveys (free-text questions) and interviews was performed. In the study, a social constructivist framework was used.^22^

During the study period of two years, fifty-nine medical students (36 female and 23 male, ages 18 to 45 years, educational semesters 2, 4, 7, 9 and 11) were included in the study and received written feedback from patients (N=189, 114 female and 75 male, 18 to 91 years).

All students in the included semesters (2, 4, 7, 9 and 11) were invited by e-mail to participate. The students and patients received oral and written information at the PHC centres. Prior to including students and patients, written consent was obtained from the management and clinical supervisors at the PHC centres. The Regional Ethical Review Board approved the study in Stockholm (Dno: EPN 2017-1574-31-1). Written consent was obtained from all participants.

### Educational context

In alignment with the generic model for doctor-patient communication at Maastricht Medical School, communication skills and patient-centredness are taught at the medical programme at KI.^3^^,^^4^ The training includes progressive skills development during workplace learning in PHC, in which the students, who are being supervised, meet patients with problems or diseases in alignment with learning goals for the respective semester. Workplace learning in PHC is included every semester, except for semesters eight and ten. The placements on average are four days each semester.

### The PFCP questionnaire

The PFCP questionnaire is a validated questionnaire developed for patients' written feedback to medical students in PHC.^20^ The questionnaire includes 19 items addressing communication and patient-centredness, using a 4-point-Likert scale with clarifying text for each scale step (from strongly disagree to strongly agree), and with alternatives 'not applicable' and 'performed by supervisor' included. After each item and at the end of the questionnaire, space for free-text comments is included.^20^

### Data collection

The data included students' evaluation survey and additional semi-structured interviews with students. The students evaluated their learning experiences from patients' written feedback obtained from the PFCP questionnaire. Data was collected by one of the authors (KB) during the students' workplace learning at PHC centres, KB had no relation to the participants in the study.

### Evaluation survey

Prior to data collection, an evaluation survey was developed to explore the students' experiences of how the patients' feedback from the PFCP questionnaire facilitated their learning regarding communication and patient-centeredness. The questions were developed through discussions among the authors. The students' evaluation survey included both open and closed questions to ensure coverage of the predefined elements.^23^ The applicability of the questions in the students' evaluation survey was tested through a pilot test, which included an overall analysis of 10 evaluation survey responses. The pilot test resulted in a few minor language adjustments. The answers from the pilot test were included in the study results.

The final evaluation survey contained nine questions (see Table 1). Three questions used a 4-point Likert scale with clarifying text for each scale step (from 'disagree strongly' to 'agree strongly'). 'Not applicable' was added as an additional option. Six questions were open-ended.

### Interview guide

The questions from the evaluation survey were also used as a semi-structured interview guide, with follow up questions added for in-depth information and examples. These questions included, for example, 'Can you further describe how you experienced receiving patients' written feedback from the PFCP questionnaire?' and 'Can you further describe how the patients' written feedback could be applied as a tool in your learning?' Areas for the follow-up questions were discussed among the authors during the process of developing the questions to the students' evaluation survey (see Table 1).^23^

**Table 1:** Students' evaluation survey including nine questions

1	How was your experience of receiving written patient's feedback?
2	The patient's feedback provided valuable information regarding my ability to apply patient-centered communication.
3	Give examples of how the patient's feedback clarified your ability to apply a patient-centered communication.
4	The patient's feedback provided guidance for future training of clinical skills.
5	Please give examples of how the patient's feedback facilitates your future training regarding clinical skills.
6	The patient's feedback helped to visualize my pedagogical assignment during the dialogue with the patient.
7	Please give examples of how, if the patient's feedback visualized your pedagogical assignment in the dialogue with the patient.
8	What was the major outcome from receiving the patient's feedback?
9	Please add if you have further comments.

### Setting and procedure

#### Patients' feedback to the student

Prior to data collection, patients gave written feedback to medical students through the PFCP questionnaire adjacent to an encounter. The patients assessed their perceived experiences with the student-led encounter regarding communication and patient-centeredness. The patients received and filled out the PFCP questionnaire in the waiting room after the student-led encounter at the PHC centre (distribution and collection of questionnaires by KB). The students were handed (by KB) the patients' feedback at the end of the workplace day during roundups with their clinical supervisor. After the students had fulfilled the clinical rotation, the PFCP questionnaires were collected, and the data was documented in an Excel spreadsheet by KB. The patients' written feedback of the perceived experience of the clinical encounter, as obtained from the PFCP questionnaire (4-point Likert scale scoring (mean, standard deviation (SD) and range) and free-text comments), is presented in Appendices 1 and 2.

### Student evaluation of learning

After receiving the patients' written feedback, obtained from the PFCP questionnaire, the students filled out an evaluation survey. In addition, to further explore the students' experiences of receiving patients' written feedback, six students participated in a semi-structured interview performed by KB. The interviews were audio-recorded and transcribed by KB. Evaluation surveys were collected and documented in an Excel spreadsheet by KB.

### Analysis

#### Quantitative data

Data from the students' evaluation survey (questions with a 4-point Likert scale) was analyzed using descriptive statistical methods (mean, SD and range)^24^ using SPSS.

#### Qualitative data

The qualitative data from the free-text questions from the students' evaluation survey and interviews were analyzed using qualitative content analysis.^25^ The free texts from the students' evaluation surveys were read repeatedly by KB and CL to obtain an overview of the students' perspectives of their learning. The meaning units were identified and condensed into the perceived key areas. The meaning units were then compared to ensure consistency. The meaning units were sorted into categories established by KB and CL.

The text from the transcribed interviews was read repeatedly for a global understanding by KB and CL, and notes were taken. The meaning units were identified. The units were condensed according to the perceived key content areas. The units were compared to ensure consistency. The meaning units were sorted into categories and established by KB and CL.

Finally, the analyses of the students' evaluation surveys and interviews were merged, resulting in four themes. All of the authors identified the themes in the process of negotiated consensus.

## Results

### Quantitative data

The results from the questions with a 4-point Likert scale in the students' evaluation survey (M, SD and range) are presented in Table 2. Due to questions not answered by the students, questions two and four had two internal dropouts, and question six had eleven internal dropouts.

**Table 2:** Descriptive statistics for questions with a 4-point Likert scale in the students' evaluation survey (N = 57)

No	Questions	Mean	SD	Range
2	The patient's feedback provided valuable information regarding my ability to apply patient-centered communication.	3.3	0.74	2 – 4
4	The patient's feedback provided guidance for future training of clinical skills.	3.2	1.31	1 – 4
6	The patient's feedback helped to visualize my pedagogical assignment during the dialogue with the patient.	3.0	1.68	1 – 4

### Qualitative data

**Figure 1:**
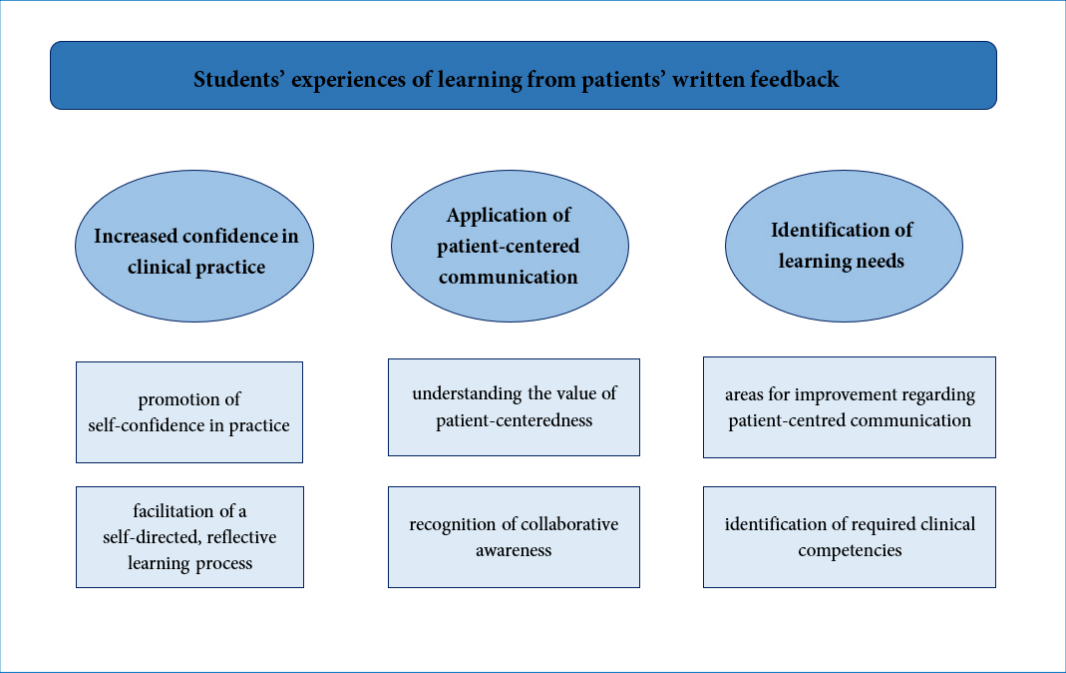
The three themes and sub-themes regarding the students' learning experiences from patients' written feedback

The qualitative analysis of the free-text answers in the students' evaluation survey and the interviews resulted in three major themes: (1) increased confidence in clinical practice, (2) application of patient-centered communication, and (3) identification of learning needs. Within each theme, two sub-themes were identified. The themes and sub-themes are illustrated in Figure 1.

### Increased confidence in clinical practice

The major content in this theme included aspects of how the patients' feedback facilitated students' confidence in their own performance. The students described that being acknowledged by the patients in certain aspects provided an increased self-confidence in clinical practice and facilitated their ability to apply a self-directed and reflective learning process.

### Promotion of self-confidence in practice

The patients' feedback was believed to confirm the adequacy of the chosen strategy and performance during the encounter. This constructive confirmation boosted the overall self-confidence in clinical practice for the students and strengthened their confidence in their present level of competence.

"The positive feedback that strengthens my self-confidence". (No 1, Female, semester 7, age 25–31)

The student's own experiences of their performance in communication and clinical competencies during the encounter were supplemented through the patients' perspectives.

"A mini-confirmation that I can capture the patient's concerns, expectations and that they experience that I saw them". (No 2, Female, semester 4, age 32–38)

### Facilitation of a self-directed, reflective learning process

In addition to self-confidence, the students indicated that the patients' feedback provided tools to facilitate a self-directed and reflective learning process.

"I saw that I sometimes did not lift and alleviate the patient's concern/dissatisfaction and received a chance to see that when I put more focus on those aspects, the patients became more satisfied". (No 3, Female, semester 11, age 18–24)

"I can link it [feedback] directly to what I did in the room, in the situation it happened, how I thought and what I can develop further". (No 4, Female, semester 7, age 18–24)

The patients' feedback also contained valuable confirmation that the students were perceived as demonstrating empathy and respect for the patients during the encounter.

"They [patients] gave positive feedback regarding how my compassion was perceived, which is difficult to estimate on my own". (No 5, Male, semester 7, age 25–31) 

"Very interesting to both being confirmed what I thought went well and rewarding/educational to realize where I did not interpret 100% of the patient's expectations right". (No 6, Male, semester 7, age 18–24)

### Application of patient-centeredness

This theme includes perspectives of how the patients' feedback clarified the value and usability of patient-centered communication as a working method in the dialogue with the patient and in the importance of the patients as a collaborative partner during an encounter.

### Understanding the value of patient-centeredness

The students described how the patients' feedback clarified the benefits of using and learning patient-centered communication techniques as a structure in a consultation.

"To continue using a patient-centered consultation and understand the value of bringing forth patients' ideas, concerns and expectations". (No 7, Female, semester 11, age 18–24)

"It was very useful to consider how my own conception of consultation was consistent with the patients'. Patients will immediately answer if they have been given the opportunity to express ideas, concerns, and expectations". (No 8, Female, semester 9, age 18–24)

### Recognition of collaborative awareness

For the students, the patients' feedback highlighted the importance of the patient as a collaborative partner during an encounter. Several students stated that the patients' feedback added a valuable perspective to better understand the power of integrating the patients' agenda, that is, the received information as a prerequisite for the provided information throughout the encounter. This also includes patient safety aspects in terms of communication techniques to secure mutual agreement and understanding. For a health provider, this emphasized the importance of having a respectful role as both the receiver and sender of information.

"Above all, getting patients' ideas, concerns, and expectations make my own work as clinician easier when I understand the patient's agenda". (No 9, Male, semester 11, age 25–31)

"A little surprising how different caregiver and patient can feel after a patient visit from a positive point of view". (No 5, Male, semester 7, age 25–31)

### Identification of learning needs

The theme includes different aspects of how the patients' subjective feedback facilitated the students' identification of specific areas and competencies for improvement.

#### Areas for improvement regarding patient-centred communication

For the students, the patients' feedback identified areas of improvement for further clinical practice regarding patient-centred communication techniques. The patients' feedback also clarified that the students must adjust their own professional language to enhance the patients' ability to understand and participate in the dialogue during the encounter.

"Suggested several shortcomings, e.g., in patient's ability to express his concerns, etc.". (No 10, Male, semester 11, age 25–31)

"Do not forget ICE [ideas, concerns and expectations]! ...". (No 11, Female, semester 7, age 18–24)

"Need to remember to ask the patient if he/she is concerned about something". (No 12, Female, semester 7, age 18–24)

In addition, the patients' feedback provided the students with valuable information, highlighting the student's pedagogical assignment throughout an encounter to provide valuable and actionable information to the patient.

"Adapt my language according to the different patients need. Be clear and avoid using medical language during the dialogues with patients". (No 13, Female, semester 11, age 25–31)

"Provides insights on whether you, as a clinician also has a pedagogical approach…". (No 14, Male, semester 4, age 18–24)

### Identification of required clinical competencies

The students experienced that the patients' feedback provided information regarding the level of clinical competences, that is, the areas for improvement of clinical knowledge and skills. The feedback also targeted the importance of the fact that to perform a patient-centred encounter, adequate theoretical and clinical knowledge is required. Many students described this as an eye-opener, stating that the combination of communication skills and medical expert knowledge is a prerequisite for a successful outcome in clinical practice.

"Clarifying warning flags for which the patient should seek medical care". (No 15, Female, semester 5, age 18–24)

"Reminded me of what I forgot to perform or ask during the medical history/clinical examination". (No 16, Male, semester 7, age 18–24)

"It becomes clear that when one feels the medical knowledge is lacking, communication becomes more restricted from my side". (No 17, Female, semester 9, age 18–24)

## Discussion

Our study explored students' learning experiences from patients' written feedback, obtained using the PFCP questionnaire, adjacent to a patient encounter.

The results from the students' experiences of learning, obtained from the students' evaluation survey (questions with a 4-point Likert scale), indicated that the patients' written feedback visualized the students' ability to apply patient-centred communication and provided guidance for further clinical training. In addition, the patient's written feedback visualized the value of the student's pedagogical assignment during an encounter. The student's experience of their learning by use of the PFCP questionnaire was explored in an evaluation survey and through semi-structured interviews, which in thematic analysis resulted in three themes: (1) increased confidence in clinical practice, (2) application of patient-centered communication, and (3) identification of learning needs.

The students in our study described that they experienced enhanced self-confidence in clinical practice as a result of patients' written feedback, which also has been described by the previous authors.^21^^,^^26^^,^^27^ Previous research also shows that patients' written feedback facilitates a self-directed reflective learning process for continuous learning, which is essential to develop knowledge, attitude and skills required for reflective professional practice.^7^^,^^15^^,^^28^^,^^29^

The usefulness of a patient-centred communication model as a tool to structure the dialogue and increase the patient's participation in the dialogue was perceived to be clarified through the patients' written feedback. The students expressed an understanding of how lack of theoretical and/or practical competencies affected both their ability to communicate and apply patient-centredness throughout the encounter. The relationship between the necessity of solid knowledge and skills and the ability to communicate and apply patient-centeredness has been described in the studies by Finch and colleagues^27^ and Lai and colleagues^30^ as essential competences for a future doctor to possess.^3^^,^^4^ The patients' written feedback also illustrated the necessity of incorporating the patients' ideas, concerns and expectations as a common ground for bilateral understanding throughout the patient encounter.

Our results of students learning from patients' written feedback are consistent with the findings in previous work despite variations in the methodology and research.^27^^,^^31^^,^^32^  The students in our study considered the patients to be knowledgeable facilitators of the students' learning process, which aligns with the results from Oswald and colleagues^32^ and Lucas and colleagues.^17^ The exploration of the patient's perceived experience, and student's own understanding of the performed encounter was found to facilitate the student's self-directed learning.^33^^-^^34^ This educational process aligns with learning within a social constructivist framework.^22^

Hattie and colleagues^15^ describe the importance of direct and concrete feedback for students to identify their own level of competence for further clinical training. Despite the small variations in the patients' ratings per item in the PFCP questionnaire, the students in our study experienced that they were provided with an actionable, concrete, interpretable and contextualized substrate, targeting specific knowledge and skills gaps that facilitated clinical learning.

Some previous studies have indicated that students prefer feedback from clinical supervisors.^26^ Only a few students in our study stated that they would rather receive feedback only from their clinical supervisors. The patients can, of course, not be the main assessor of the student's level of competence and are usually not familiar with the learning goals and milestones in medical education. However, as the content of the PFCP questionnaire is in alignment with the common framework for patient-centred communication, the feedback could be regarded as a valuable tool for patients' written feedback in students' workplace learning.^3^^,^^4^ Therefore, the patients' feedback could perhaps also be applied as an additional source in the assessment of medical students' competence. Further research is required to explore the field of patients' written feedback as a tool for assessing competence in medical education.

### Strengths and limitations

The current study included students from several semesters at eight PHC centres representing different socioeconomic populations. The patients further represented different ages and genders with various problems and diseases. Data from the students' evaluation surveys were further elaborated with the addition of individual interviews to capture in-depth perspectives regarding the students' experiences of their own learning. The detailed description of the collection and analysis of the data aimed to increase the transferability of our findings. The cohesion of the results in the students' evaluation surveys and interviews and the results of the descriptive data indicated that a mixed-methods approach for data collection was appropriate.

Although the findings in this study suggest that written feedback from patients obtained directly after an encounter can play a more significant role in medical education, there are some limitations to consider. One such limitation could be that the present study only considered students' experiences of patients' written feedback from a specific questionnaire, which could limit the generalisability of our findings. The questionnaire focused on communication skills and patient-centredness, hence potentially limiting the patients' opportunity to provide feedback from other perspectives. To compensate for an eventual lack of aspects that were not perceived to be covered by the items, the patients were offered to add free-text comments after each item and in a supplementary open-ended last question.

During the present study, some questions were answered, and a number of questions remain to be explored within the field of patients' written feedback as a facilitator in students' workplace learning. Further studies are also required regarding the models for implementing the patients' written feedback in different educational or clinical contexts. In addition, further studies are required to explore how patients' scoring items in the PFCP questionnaire can be related to the fulfilment of the intended learning outcomes.

## Conclusions

The results indicate that patients' written feedback obtained from the PFCP questionnaire can be an important resource in students' workplace learning and an adequate educational tool facilitating communication and patient-centredness learning in medical education. Through the PFCP questionnaire application, which provided concrete feedback adjacent to a patient encounter, students' motivation for further clinical training, progressive development of communication skills and the application of patient-centredness could be facilitated. The patients' feedback was perceived as a useful resource for the students to identify the ability to communicate and apply patient-centredness during an encounter and to target areas for further clinical training during the students' workplace learning. The patients' feedback seemed to provide students with increased confidence in clinical practice, which facilitated a reflective, self-directed learning process. Furthermore, patient-centeredness was stressed as an important working method, and increased awareness of the patient as a collaborative partner throughout an entire encounter was visualized through the patients' feedback. The patients' written feedback adjacent to a patient encounter was identified as a valuable additional learning tool in medical students' workplace learning. Further studies are required to explore how patients' written feedback can be operationalized in different clinical contexts, for example, within in-patient care.

### Acknowledgements

We gratefully acknowledge the heads of the PHC centres, the clinical supervisors, and the staff at PHC centres and the medical students and the patients for their participation and for making this study and subsequent article possible.

### Conflict of Interest

The authors declare that they have no conflict of interest.

## Supplementary material

Supplementary file 1 Appendix 1. The Patient's Feedback in Clinical Practice (PFCP) questionnaire, including descriptive statistics, for patients (N = 189) feedback to medical students (N = 59)

Supplementary file 2 Appendix 2. The results from the qualitative content analysis of the patients' free-text comments from the PFCP questionnaire
