# Supplementary material for: Learning from patients' written feedback: medical students' experiences
Source: Int J Med Educ. 2022 Jan 31;13:19–27. doi: 10.5116/ijme.61d5.8706 (PMC9017500; doi:10.5116/ijme.61d5.8706)
Supplement: Supplementary file 1 — Appendix 1. The Patient's Feedback in Clinical Practice (PFCP) questionnaire, including descriptive statistics, for patients (N = 189) feedback to medical students (N = 59) [file ijme-13-19-S1.pdf]

## Appendix 1

The Patient's Feedback in Clinical Practice (PFCP) questionnaire, including descriptive statistics, for patients (*N* = 189) feedback to medical students (*N* = 59)

| No | The PFCP questionnaire questions | Mean | SD | Range |
|---|---|---|---|---|
| 1. | Did you have the opportunity to explain the reason for your visit or what had happened since you last visited the doctor? | 3.80 | 0.74 | 3 – 4 |
| 2. | Did you have the opportunity to explain your own thoughts regarding your problems? | 3.81 | 0.68 | 1 – 4 |
| 3. | Did you have the opportunity to explain if there was something that worried you regarding your problems? | 3.66 | 0.98 | 1 – 4 |
| 4. | Did you have the opportunity to express if there was something specific you wanted to be performed/initiated during the consultation? | 3.51 | 1.2 | 1 – 4 |
| 5. | Did the student confirm with you that he/she understood your cause of concern correctly by summarising what you told him/her? | 3.69 | 0.94 | 2 – 4 |
| 6. | Did the student explain his/her medical questions, so you understood why they were asked? | 3.22 | 1.38 | 1 – 4 |
| 7. | During the clinical examination, did the student explain why certain examinations were performed? | 3.30 | 1.31 | 1 – 4 |
| 8. | Did the student take into consideration your own thoughts regarding your problem when you discussed the follow-up plan/treatment? | 3.33 | 1.44 | 1 – 4 |
| 9. | Did you receive information/explanation from the student which made it possible for you to participate in the planning of care/treatment? | 3.13 | 1.54 | 1 – 4 |
| 10. | Did the student provide information about suggested care/treatment in a way that you understood? | 3.01 | 1.68 | 2 – 4 |
| 11. | Did the student provide information about medication in a way that you understood? | 1.89 | 1.96 | 2 – 4 |
| 12. | Did the student provide information in a way that you understood regarding symptoms that call for immediate contact with healthcare? | 2.11 | 1.88 | 1 – 4 |
| 13. | Did the student ask if the information you were given was interpretable? | 3.21 | 1.53 | 1 – 4 |
| 14. | Did you have the opportunity to bring up questions you had before the visit regarding your cause of concern? | 3.33 | 1.44 | 2 – 4 |
| 15. | Did the student involve you in the decision-making process regarding your care/treatment? | 3.07 | 1.61 | 2 – 4 |
| 16. | Were you involved in the decision-making process regarding your care/treatment to the extent you wanted? | 3.19 | 1.53 | 2 – 4 |
| 17. | Are you satisfied with the initial plan that was decided upon together with the student? | 3.39 | 1.38 | 1 – 4 |
| 18. | Did you experience that the student treated you with compassion and consideration? | 3.91 | 0.47 | 2 – 4 |
| 19. | Did you experience that the student treated you with respect and dignity? | 3.95 | 0.33 | 3 – 4 |
