# Supplementary material for: Learning from patients' written feedback: medical students' experiences
Source: Int J Med Educ. 2022 Jan 31;13:19–27. doi: 10.5116/ijme.61d5.8706 (PMC9017500; doi:10.5116/ijme.61d5.8706)
Supplement: Supplementary file 2 — Appendix 2. The results from the qualitative content analysis of the patients' free-text comments from the PFCP questionnaire [file ijme-13-19-S2.pdf]

Appendix 2

The results from the qualitative content analysis of the patients' free-text comments from the PFCP questionnaire

Clinical examination skills	Request for additional information	Positive affirmation, with concrete example	Targeting the patient's agenda
She was verbal, communicative throughout the encounter and had an easy touch while examining.	*Please, tell me why you listen to my heart and maybe what you are searching for.*	*Very good questions, asked in a calm pace.*	*Received answers regarding my cause of concern.*
Listened to my lungs so gently.	*You could explain why I should perform certain movements with my arms. What is it you are looking for?*	*Good and attentive listener. Took time to hear me out.*	*Satisfied with the encounter and what it resulted in.*
The clinical examination was conducted with knowledge and compassion, e.g., warmed the hands before the examination.		*Superb encounter! The feeling of been taken completely seriously.*	*Went through my problem with thoroughness.*
		Good demeanour during the encounter, explained carefully.	
